# Precocious reproduction increases at the leading edge of a mangrove range expansion

**DOI:** 10.1002/ece3.2270

**Published:** 2016-06-26

**Authors:** Emily M. Dangremond, Ilka C. Feller

**Affiliations:** ^1^ Smithsonian Environmental Research Center 647 Contees Wharf Road Edgewater Maryland 21037

**Keywords:** Life history, local adaptation, mangrove, range expansion, *Rhizophora mangle*

## Abstract

Climate change‐driven shifts in species ranges are ongoing and expected to increase. However, life‐history traits may interact with climate to influence species ranges, potentially accelerating or slowing range shifts in response to climate change. Tropical mangroves have expanded their ranges poleward in the last three decades. Here, we report on a shift at the range edge in life‐history traits related to reproduction and dispersal. With a common garden experiment and field observations, we show that *Rhizophora mangle* individuals from northern populations reproduce at a younger age than those from southern populations. In a common garden at the northern range limit, 38% of individuals from the northernmost population were reproductive by age 2, but less than 10% of individuals from the southernmost population were reproductive by the same age, with intermediate amounts of reproduction from intermediate latitudes. Field observations show a similar pattern of younger reproductive individuals toward the northern range limit. We also demonstrate a shift toward larger propagule size in populations at the leading range edge, which may aid seedling growth. The substantial increase in precocious reproduction at the leading edge of the *R. mangle* range could accelerate population growth and hasten the expansion of mangroves into salt marshes.

## Introduction

Species distributions are dynamic, responding on multiple time scales to shifts in climate and biotic interactions. Species undergoing range expansions encounter novel ecological conditions at the expanding range edge, including low‐density conspecifics, a lack of natural enemies, new climatic conditions, and altered competitive interactions. During a range expansion, these factors can affect life‐history traits such as population growth rate, dispersal, and competitive ability (Chuang and Peterson 2016). Many newly founded populations display shifts in a variety of traits. In a variety of taxa, individuals in new populations reach sexual maturity faster, such as *Prosopis* spp. [mesquite] (Pasiecznik et al. 2006), *Rhinella marina* [cane toads] (Phillips 2009), *Coregonus albula* [vendace] (Amundsen et al. 2012), and *Lythrum salicaria* [purple loosestrife] (Colautti and Barrett 2013). A shift toward earlier reproduction could increase population growth at the range edge, hastening the spread of an invasion or range expansion.

**Figure 1:**
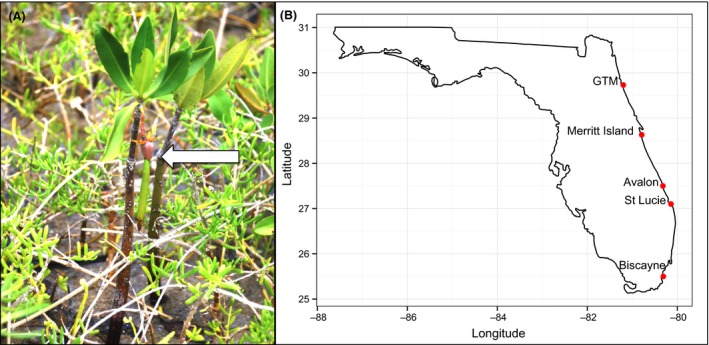
(A) Precocious reproduction in *Rhizophora mangle*. A 2‐year‐old *R. mangle* seedling is shown here with a propagule forming. (B) Locations of source populations for common garden experiment. Common garden was placed at 30.0°N, 81.3°W.

Mangroves are woody plants living in the intertidal zone in tropical and subtropical climates. Currently, mangrove species are expanding their ranges poleward into higher latitude temperate salt marshes and increasing in abundance near their northern range limits (Rogers et al. 2005; Osland et al. 2013; Cavanaugh et al. 2014). This increase in mangroves may have a positive impact on ecosystem services such as increased carbon storage (Chmura 2003) and an increased ability to control flooding from tropical storms (Krauss et al. 2009), but also can displace salt marsh plants and disrupt salt marsh food webs, which could have negative consequences for fisheries and wildlife (Glick and Clough 2006). Mangroves have doubled in abundance in the mangrove–salt marsh ecotone in northern Florida in the past 28 years, coincident with fewer severe freezes (Cavanaugh et al. 2014). We have observed precociously reproducing mangroves at the leading edge of the mangrove invasion in northern Florida. In this area, we have documented numerous incidences of seedlings of *Rhizophora mangle* L. that have begun reproducing in the first one to 2 years after establishment (Fig. 1). The purpose of this study was to determine the degree to which precocious reproduction varies among *R. mangle* populations as they approach their northern range limit on the Atlantic coast of Florida. We examined reproductive traits of *R. mangle* along a latitudinal gradient on the Atlantic coast of Florida with a common garden experiment and field observations. Based on initial observations, we hypothesized that precocious reproduction would occur at a higher rate in northern populations compared to southern populations.

## Methods

Mangroves are restricted to the intertidal zone in tropical and subtropical climates and produce viviparous propagules that are water‐dispersed. Propagules are different from seeds because they do not go dormant and are essentially an unrooted seedling. Propagules of *R. mangle* consist of a long cigar‐shaped hypocotyl that detaches from the fruit and can start rooting after a short floating period (Tomlinson 1986). In Panama, *R. mangle* propagules have a mass of 14 g and are 22.1 ± 1.0 cm long (Rabinowitz 1978). In the United States, mangroves reach their northernmost limit in St. Augustine, FL (30.0°N, 81.3°W). North of 30°N, salt marsh vegetation dominates the intertidal zone and south of 28°N, and mangroves are the dominant vegetation in the intertidal zone. In between 28°N and 30°N, mangroves and salt marsh co‐occur in an ecotone. Our study areas are on the Atlantic coast of Florida and include sites in the pure mangrove zone in southern Florida and in the mangrove–salt marsh ecotone in central to northern Florida. Between 1984 and 2011, the spatial extent of mangroves more than doubled between latitude 29 and 29.75°N, increasing from 605 ha to 1302 ha. During the same period, the mangrove spatial extent between 27°N and 28°N increased from 3409 ha to 4019 ha (Cavanaugh et al. 2014).

### Field observations

In naturally occurring populations, three sites were sampled to determine age at first reproduction of *R. mangle*: at the northern range limit (29.7°N, 81.2°W), in the middle of the mangrove–salt marsh ecotone (29.1°N, 80.9°W), and south of the ecotone in the pure mangrove zone (27.5°N, 80.3°W). In the northern and middle sites, all *R. mangle* individuals were measured along three 100‐m transects. South of the ecotone where *R. mangle* is more abundant, all plants within a 1‐m^2^ plot were measured, with plots sampled every 10 m along three 100‐m transects. Reproductive stems of *R*. *mangle* leave a distinctive scar, allowing us to score a plant as reproductive or nonreproductive, and calculate the age at first flowering based on the number of leaf nodes produced prior to a flowering node. By observing plants in the common garden and in the shadehouse, we know *R. mangle* individuals produce four to five pairs of leaf nodes per year, so we were able to age *R. mangle* seedlings and saplings based on the number of leaf nodes present. This technique has also been used for this species in Panama (Duke and Pinzón 1992). We estimated age of first reproduction by dividing the number of nodes by five, then rounding up; this means that seedlings with flower scars at node nine or 10 would both be called reproductive by age 2, but a plant with a reproductive scar at node 11 would be called reproductive by age 3. We used time‐to‐event analysis to test for differences in time to reach reproductive maturity. Data were right‐censored, meaning not all individuals have reached reproductive maturity.

Propagule size does not affect seedling survival, but can affect seedling growth (Lin and Sternberg 1995; Sousa et al. 2003). To examine latitudinal patterns of propagule size, we collected propagules of *R. mangle* from additional populations on the east coast of Florida in September 2014 and measured length from the base of the propagule to the cotyledonary collar. Flowering occurs over a several month period (June–August), and fertilization and propagule development are staggered throughout the season. A random sample would have included propagules of different ages, so the 30 largest propagules were collected from each site, with no more than three propagules from the same individual. To test how latitude affected propagule size, we fitted a linear regression, linear regression on log‐transformed data, and a polynomial regression to the means for each site, and the best fitting model was chosen with Akaike's information criterion (AIC).

### Common garden

**Figure 2:**
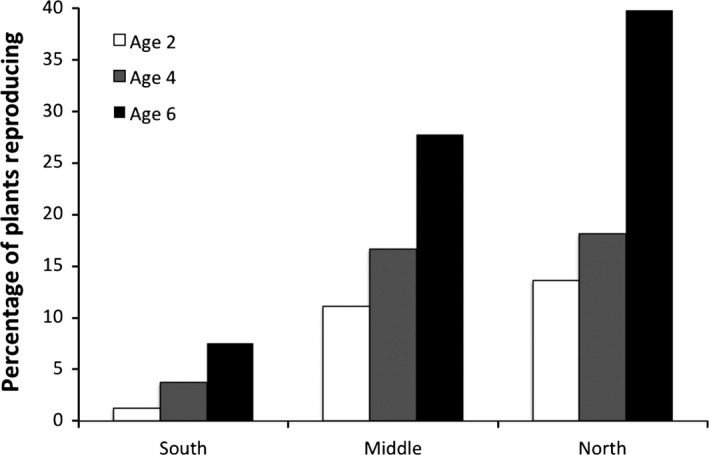
Percentage of *Rhizophora mangle* individuals reproducing by ages 2, 4, and 6 in field populations south of the mangrove–salt marsh ecotone, in the middle of the ecotone, and at the northern end of the ecotone. The mangrove–salt marsh ecotone on the Atlantic coast of Florida occurs between ~28 and 30°N.

We established a common garden in December 2012 at the Guana‐Tolomato‐Matanzas National Estuarine Research Reserve in St. Augustine, Florida (30.0°N, 81.3°W). The common garden was located ~75 m from the water's edge in a salt marsh zone dominated by *Spartina alterniflora*. Interstitial soil salinity ranged from 30 to 35 psu. In late September and early October 2012, propagules of *R. mangle* were collected from five sites spanning the Atlantic coast of Florida (Fig. 2). The sites were as follows, from south to north: Biscayne Bay National Park (25.5°N, 80.2°W), St Lucie Inlet State Park (27.1°N, 80.1°W), Avalon State Park (27.5°N, 80.3°W), Merritt Island National Wildlife Refuge (28.7°N, 80.7°W), and Guana‐Tolomato‐Matanzas National Estuarine Research Reserve (29.7°N, 81.2°W). At each site, 40–50 propagules were randomly collected from trees, with no more than three per tree. Propagules were planted in topsoil in individual tree tubes at the Smithsonian Marine Station in Fort Pierce, Florida, and were grown in seawater in an outdoor shadehouse for 2 months until transfer to the common garden in December. When transplanted into the common garden, we removed seedlings from their tubes and inserted them directly into the ground. At the time of transplanting, seedlings had one pair of leaves and had grown an average of 1.6 cm (±0.05) above the hypocotyl. We planted seedlings in three blocks separated by 10 m. Each block contained six 1‐m squared plots into which we planted 13 seedlings. Seedlings were randomized so at least two from each source population were planted into each plot. We monitored seedlings for survival, growth, and reproductive status at 3, 10, 14, 20, and 32 months after planting. We analyzed growth after 32 months with a one‐way analysis of variance, and we tested for differences in survival curves among populations with a log‐rank test. We analyzed flowering along the latitudinal gradient with a logistic regression.

## Results

### Field observations

**Figure 3:**
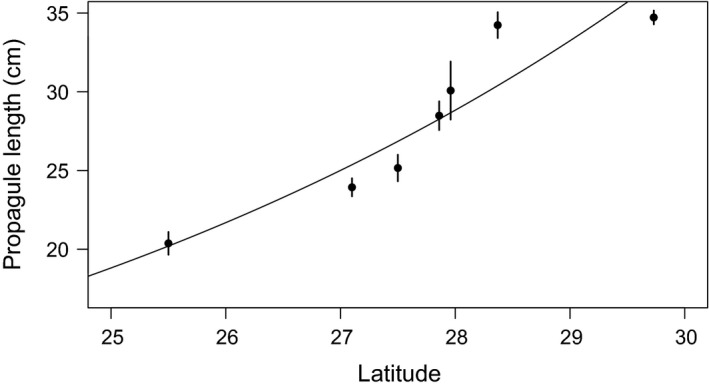
*Rhizophora mangle* propagule length increases with latitude in sites spanning the Atlantic coast of Florida. The 30 largest propagules were collected from each site, with no more than three propagules from the same individual. Points are means ± SE for each site. The fitted line is *y* = e^(−0.622 + 0.142*x*) (*r* ^*2*^ = 0.878).

**Figure 4:**
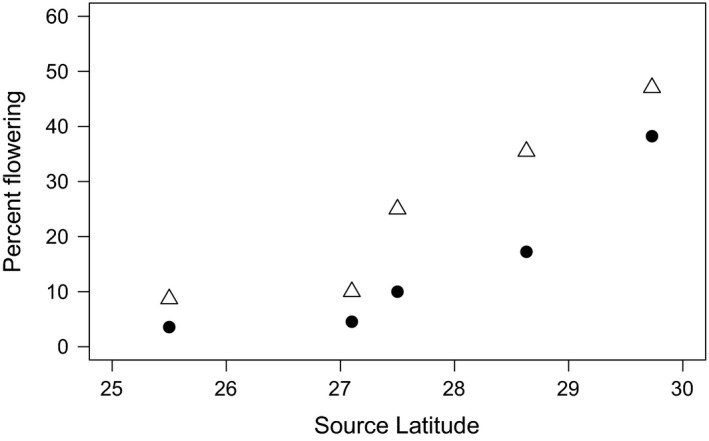
Percentage of flowering 2‐year‐old (circles) and 3‐year‐old (triangles) *Rhizophora mangle* seedlings planted in a common garden at 30°N.

We sampled *R. mangle* in naturally occurring populations and found that there is a significant difference among sites in the age at which individuals reach reproductive maturity (log‐rank test, *X* ^2^ = 9.5, df = 2, *P* = 0.008). Individuals from pure mangrove sites south of the ecotone are much less likely to be reproductive by age 6 than individuals in the middle of the ecotone or at the northern range edge (Fig. 3). Additionally, mangrove propagule size changes along the mangrove–salt marsh ecotone, with *R. mangle* propagules approaching larger sizes with increasing latitude. Mean propagule length was related linearly to latitude on a log scale (Fig. 4, *F*_1,5_ = 36, *P* = 0.002).

### Common garden experiment

In a common garden at the northern range limit of *R. mangle* (30°00′46″ N, 81°20′40″W), the proportion of *R. mangle* seedlings that flowered in their second year increased with latitude of the source population (logistic regression, odds ratio = 2.16, [95% CI = 1.45–3.49], *P* = 0.001). By year three, overall flowering increased compared to the previous year (Fig. 3), but the pattern of flowering increasing with latitude was maintained (logistic regression, odds ratio = 1.77, [95% CI = 1.31–2.51], *P* = 0.001). Seedling survival in the common garden was not significantly different among source populations (log‐rank test, *R. mangle*:* X* ^2^ = 4.3, df = 4, *P *= 0.361), with 40–60% of each population surviving to 3 years. *Rhizophora mangle* seedling growth was significantly different among source populations (analysis of covariance [ANCOVA], df = 4, *F* = 14.33, *P* = <0.001), with seedlings from the northernmost population growing 4 cm less, on average, than seedlings from other populations. Growth over the previous year did not differ significantly between plants that flowered and those that did not flower (*t*‐test, *t* = −0.47, df = 81.96, *P*‐value = 0.639). Additionally, initial propagule size did not affect growth rates over 3 years (ANCOVA, df = 1, *F* = 0.114, *P* = 0.736).

## Discussion

Individuals from populations at the northern range limit of *R. mangle* reproduce at a younger age than individuals from populations further south. A previous study (Proffitt and Travis 2014) found a positive relationship between cold stress and flowering in *R. mangle*, but used latitude as a proxy for cold stress. Therefore, it is unknown whether plants from higher latitudes are flowering in response to cold or because of population‐level differences. Our common garden experiment shows the increased rate of flowering in individuals from the northern end of the range is not explained solely by cold stress because in the common garden, all seedlings experienced the same conditions.

The reason for the high rate of precocious reproduction in northern populations is unknown, but based on our common garden results, it is likely that there is a genetic basis to precocious reproduction. We did not explicitly test for maternal effects, but Proffitt and Travis (2014) showed that maternal line influenced reproduction in *R. mangle* seedlings on the Gulf Coast of Florida. Their results, taken together with ours, suggest that in this species, there are early‐reproducing genotypes and that these early reproducers are more prevalent in the northernmost populations. The reason for this prevalence of early reproducers is unclear. One explanation is that the northern populations were colonized by early‐reproducing genotypes through a founder effect. However, this does not explain the gradual increase in early reproducers in populations south of the ecotone. Another explanation is that northern populations were colonized by all genotypes, but the early‐reproducing genotypes spread their genes more quickly through the population via increased reproductive output. A third explanation is that local adaptation has created the latitudinal gradient in precocious reproduction. Individuals that can reproduce early in life have higher fitness compared to later‐reproducing individuals; in sites where freezes occur, early reproducers would be selected, creating a cline in reproductive timing. There may have been a preexisting cline that we are only now detecting because of the recent rapid population growth at the northern range edge.

An alternative explanation is that precocious reproduction may result from inbreeding. Inbreeding led to precocious reproduction in offspring of *Pinus banksiana*,* Crepis tectorum*, and invasive populations of *Spartina alterniflora*. Precocious reproduction is heritable in some trees, including *Pinus sylvestris*,* Betula verrucosa,* and *B. pubescens*. *Rhizophora mangle* is self‐compatible and usually wind‐pollinated. Plants at the leading edge of the range are more likely to be solitary (Zomlefer et al. 2006; Williams et al. 2014), which may force them to self‐pollinate.

In addition to precocious reproduction, propagule size of *R. mangle* also increases with increasing latitude. Propagule length is correlated with propagule mass, so longer propagules also have a larger biomass (Lin and Sternberg 1995). Establishment rates of *R. mangle* do not vary with propagule size, but larger propagules develop into larger seedlings with longer shoots and more leaves (Sousa et al. 2003) and are able to fix more carbon than seedlings developing from smaller propagules (Lin and Sternberg 1995). This could provide a competitive advantage as seedlings establish and also may allow the seedling to allocate energy to reproduction at an early age. The consequences of this size increase for dispersal are unknown, but *R. mangle* propagules remain viable for months after floating (Rabinowitz 1978), so the increased maternal reserves may aid propagules in longer dispersal periods.

Under a dense canopy in Panama, *R. mangle* individuals often take 10–15 years to reproduce (WP Sousa, pers. comm.), and even plants growing in full‐sun tidal fringe in Belize never flowered before the age of 4 (Farnsworth et al. 1996). Although they do not give an age at first flowering, Gill and Tomlinson (1969) state that flowering in *R. mangle* in southern Florida begins on saplings when they are more than a meter tall. Our plants were 40–50 cm tall when they began flowering, so this is a surprising life‐history shift. To our knowledge, such a shift in life‐history traits in a native species undergoing a range expansion has not been documented.

Historically, the limits to species ranges were attributed to abiotic factors such as temperature and precipitation. While their contributions to the recent range expansion are undocumented, precocious reproduction and the potential for increased dispersal in *R. mangle* are two biotic factors that may contribute to increases in abundance in northern populations and future range expansion. Rapid changes in demographic traits are increasingly recognized as important for the success of a species invading a new environment. A younger age at first reproduction increases the intrinsic rate of population growth (Cole 1954) and has contributed to the success of non‐native species invading new areas (e.g., *Spartina alterniflora* [Davis 2005], *Prosopsis* spp. [Pasiecznik et al. 2006], *Rhinella marina* [Phillips 2009], and *Coregonus albula* [Amundsen et al. 2012]). Many examples exist of invasive species growing faster in their introduced range than in their native range (e.g., Leishman et al. 2000; Phillips 2009), but fewer examples exist of a shift in reproductive traits in native species expanding their ranges.

Mangroves provide ecosystem services valued over $190,000 ha/year (Costanza et al. 2014), but their expansion comes at the expense of coastal salt marshes. The consequences of the conversion of salt marsh habitat to mangrove habitat are unknown, but will likely result in profound ecosystem level changes. These changes include a shift from an ecosystem dominated by herbaceous plants to one dominated by woody plants, an increase in carbon storage (Kelleway et al. 2015; Doughty et al. 2016), and shifting habitat use by mangrove and salt marsh associated animals (Riley et al. 2014). The number of *R. mangle* individuals in the northernmost population has increased from ~10 in 2005 (Zomlefer et al. 2006) to ~100 found in this study. Mangroves are predicted to move northward in Florida at a rate of 2.2–3.2 km/yr over the next 50 years due to climate change (Cavanaugh et al. 2015). The precocious reproduction demonstrated in this study may have contributed to rapid population growth of *R. mangle* in the last decade and might also result in an increase in *R. mangle* abundance as the species continues to expand its range northward.

## Conflict of Interest

None declared.
